# Uncoupling of the Electron Transport Chain Compromises Mitochondrial Oxidative Phosphorylation and Exacerbates Stroke Outcomes

**DOI:** 10.4172/2314-7326.1000283

**Published:** 2018-12-31

**Authors:** Kimberly A Grasmick, Heng Hu, Emily A Hone, Imran Farooqi, Stephanie L Rellick, James W Simpkins, Xuefang Ren

**Affiliations:** 1 Department of Neuroscience, Center for Basic and Translational Stroke Research, West Virginia University, Morgantown, USA; 2 Department of Microbiology, Immunology and Cell Biology, Center for Basic and Translational Stroke Research, West Virginia University, Morgantown, USA; 3 Department of Physiology and Pharmacology, Center for Basic and Translational Stroke Research, West Virginia University, Morgantown, USA; 4 Experimental Stroke Core, Center for Basic and Translational Stroke Research, West Virginia University, Morgantown, USA

**Keywords:** Blood-Brain Barrier (BBB), Cerebral Endovascular Cells (CECs), Carbonyl Cyanide-4 (trifluoromethoxy) Phenylhydrazone (FCCP), Electron Transport Chain (ETC), Ischemia, Transient Middle Cerebral Artery Occlusion (tMCAO), Triphenyl Tetrazolium Chloride (TTC)

## Abstract

**Objective:**

Mitochondrial dysfunction is known to be implicated in stroke, but the complex mechanisms of stroke have led to few stroke therapies. The present study to disrupted mitochondrial oxidative phosphorylation through a known electron transport chain (ETC) uncoupler, Carbonyl cyanide-4 (trifluoromethoxy) phenylhydrazone (FCCP). Analyzing the resulting neurological deficits as well as infarct volume could help determine the role of mitochondria in stroke outcome and determine whether uncoupling the ETC could potentially be a strategy for new stroke therapies. The objective of this study was to determine the effects of uncoupling electron flow on mitochondrial oxidative phosphorylation and stroke infarction.

**Methods:**

Cerebral endovascular cells (CECs) were treated with various concentrations of FCCP, and bioenergetics were measured. For the stroke mouse model, FCCP (1 mg/kg, i.p) or vehicle was administered followed by 1-hour transient middle cerebral artery occlusion (tMCAO). Infarct volume was measured after a 23-hour reperfusion, and triphenyl tetrazolium chloride (TTC) staining was used to assess infarct volume.

**Results:**

FCCP significantly decreased basal respiration, ATP turnover, maximal respiration, and spare capacity when the concentration of FCCP was greater than 1000 nM. The mice pretreated with FCCP had a significantly increased infarct volume within the cortex, striatum, and total hemisphere. Mice receiving FCCP had a significantly increased neurological deficit score compared to the vehicle.

**Conclusions:**

FCCP compromised mitochondrial oxidative phosphorylation in CECs in a dose-dependent manner. Uncoupling the electron transport chain with FCCP prior to tMCAO exacerbated stroke infarction in mice.

## Introduction

Stroke remains the third leading cause of death worldwide and is considered to be a leading cause of disability in adults [1]. Annually, 15 million people are affected by stroke, leaving 5 million dead and another 5 million disabled [2]. Stroke therapies are limited for patients due to the complicated mechanisms of stroke that have yet to be investigated.

Mitochondrial dysfunction is not only implicated in stroke, but other studies indicate that disruption of the electron transport chain also leads to mitochondrial associated metabolic diseases, such as obesity, neurodegenerative diseases, and cancer [3]. Mitochondrial oxidative phosphorylation is imperative to the proper functioning of aerobic cells, as it produces most of the energy by coupling respiration to ATP production [4]. Previous studies have shown five levels of mitochondrial oxidative phosphorylation regulation. These include direct modulation of the electron transport chain kinetic parameters, regulation of intrinsic efficiency, mitochondrial network dynamics, mitochondrial biogenesis and degradation, and the cellular and mitochondrial environment [5]. Mitochondrial dysfunction is known to affect stroke due to its incorporation in multiple cellular pathways. These pathways include ATP and reactive oxygen species generation, energy metabolism, cell cycle, calcium homeostasis, and apoptosis [6]. It controls the fate of neuronal cells while also influencing blood-brain barrier (BBB) permeability after the occurrence of ischemic stroke. Mitochondria heavily regulate the ETC since it is the site of redox reactions that facilitate phosphorylation of ADP to ATP. Defects in mitochondria and subsequently the ETC are known to have fatal consequences for cells.

Carbonyl cyanide-4 (trifluoromethoxy) phenylhydrazone (FCCP), a mobile ion carrier, is a known uncoupling agent of the mitochondrial electron transport chain [7]. FCCP is able to disrupt ATP synthesis through uncoupling the proton gradient generated by the mitochondrial membrane. FCCP has been associated with mitochondrial inhibition and has been shown to activate ionic currents and depolarize the plasma membrane potential in a dose-dependent manner [8, 9]. Other *in vivo* studies found that FCCP disrupts mitochondrial H^+^ gradients, disrupts the microtubular network, and decreases the rate of protein synthesis. Stroke studies have used FCCP to reduce the mitochondrial membrane potential and increase K^+^ conductance to shunt action potentials [10]. However, the role of FCCP on stroke outcomes were not reported.

In this study, we investigated the relationship between the uncoupling of the electron transport chain and infarct volume in stroke. Using an *in vitro* cell culture model, we demonstrated that FCCP compromised mitochondrial phosphorylation in a dose-dependent manner. Using a transient middle cerebral artery occlusion stroke model, we demonstrated that uncoupling the electron transport chain exacerbates stroke infarction in mice. This novel study determined that the role of uncoupling the electron transport chain is detrimental to stroke brain and targeting of the electron transport chain could potentially be a strategy for stroke therapy.

## Materials and Methods

### Animals

C57BL/6J male mice (3–6-month-old, 25~30 g) from the Jackson laboratory were used for experiments. All procedures were approved prior to experimentation by the West Virginia University Animal Care and Use Committee.

### Cell culture

The cerebrovascular endothelial cells (CECs, b. End3 cell line from ATCC) are originally derived from mouse brain endothelial cells. Passages 25~30 were used for *in vitro* experiments. The cells (16,000 cells/ well) were seeded into Seahorse Bioscience XFe96 cell culture plates overnight then treated with FCCP in varying concentrations (10 nM, 100 nM, 1000 nM, 10000 nM) or the vehicle control for 24 hours in a 37°C humidified incubator with 5% CO_2_.

### Measuring oxygen consumption in CECs

Oxygen consumption rate (OCR) was measured at 37°C using a Seahorse XFe96 Analyzer (Agilent, Santa Clara, CA) according to our protocol published [11]. Briefly, the cell culture media was changed to Mito-Stress Test Assay Media, which is DMEM that contains the following: 2 mM GlutaMax, 1 mM sodium pyruvate, and 25 mM glucose (media is un-buffered, pH 7.4). The cells were incubated at 37°C without CO_2_ 1 hr prior to the start of the extracellular flux assay. Oligomycin, carbonyl cyanide 4-(trifluoromethoxy) phenylhydrazone (FCCP), rotenone and antimycin A (all from Sigma, St. Louis, MO ) of 10 × compound dilutions were prepared for the assay and loaded into the assigned ports. A sensor cartridge was calibrated 24 hr prior to the assay. Following calibration, the cell plate was placed in the Bioanalyzer, and the Mito-Stress Test assay protocol was completed on the samples. This protocol allowed determination of the basal level of oxygen consumption, the amount of oxygen consumption linked to ATP turnover, the maximal respiration capacity, and the spare capacity.

### Drug administration

For the *in vivo* study, mice were pretreated with FCCP (Sigma, 1 mg/kg n=8) or vehicle (10% DMSO in saline) (n=8) via intraperitoneal injection 30 min prior to stroke model.

### Murine ischemic stroke model

Transient middle cerebral artery occlusion (tMCAO) was performed after induction of anesthesia with 4~5% isoflurane and mice did not respond to toe pinch. Isoflurane (1–2%) *via* face mask in 30%O_2_: 70%N_2_ mixture was used to maintain the unresponsive state, and a rectal body temperature of 37 ± 0.5 °C was maintained throughout surgery. Right middle cerebral artery was occluded for 60 minutes with MCAO suture (diameter with coating 0.22 ± 0.01 mm from Doccol Corp., MA). Blood flow and success of occlusion was determined using Laser Speckle Imager (Moor Instruments, United Kingdom).

### Brain histology

Mice were euthanized under deep anesthesia with 4~5% isoflurane; brains were retrieved and cut in 2 mm coronal matrix. Sliced brain sections underwent three different staining procedures: Triphenyl tetrazolium chloride (TTC, Sigma, Saint Louis, MO), Cresyl Violet, and H&E staining. Brain sections (2 mm) were stained for 30 minutes with 2% TTC in phosphate buffered solution (PBS) at 37°C staining to analyze infarct volume within the cortex, striatum, and total hemisphere. Resulting brain sections were imaged with photo scanner (CanonScan, 9000 F, Japan). Brain sections were fixed in 10% formalin for one week then embedded in paraffin. Coronal slices (10 μm) were cut through the brain hippocampus area. Cresyl violet [12] and H&E [13] staining were performed using published protocols. One FCCP treated mouse died before 23-hour end-point was not included for stroke infraction.

### Neurological deficits

After being subjected to tMCAO, a score was given to each mouse to evaluate neurological deficits. Neurological functioning was measured on a 0–5 scale, where 0=no neurological dysfunction; 1=failure to extend contralateral forelimb when lifted by tail; 2=circling to the contralateral side: 3=falling to the contralateral side; 4=nonspontaneous walk or in a comatose state; 5=death.

### Statistical analysis

A one-way ANOVA and post hoc Tukey’s test were used to analyze the graph and calculate basal respiration, ATP turnover, maximal respiration, and spare capacity, which are all derivatives of mitochondrial oxidative phosphorylation. Student’s t-test was used to analyze the significance of infarction and neurological deficits between groups. p<0.05 was considered statistical significance.

## Results

### FCCP compromises oxidative phosphorylation in CECs

**Figure 1:**
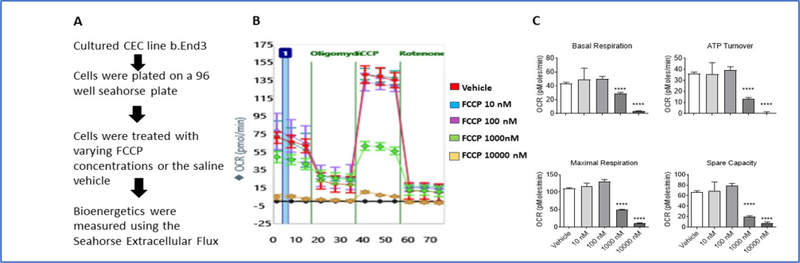
**(A-C)** Uncoupling the ETC with FCCP compromised mitochondrial oxidative phosphorylation in CECs. (A) End3 cells were exposed to varying concentrations of FCCP concentrations and a Mito-Stress test performed. (B) Oxygen consumption rate (OCR) of CECs. (C) Basal respiration, ATP turnover, maximal respiration, and spare capacity significantly decreased following FCCP exposure at 1000 nM and 10000 nM (****p<0.0001), while there was no observable decrease at the lower concentrations. Data are presented as mean ± SEM.

To assess the effects of uncoupling the electron transport chain in CECs, Seahorse XFe96 Bioanalyzer with the Mito-Stress Test was utilized. Briefly, the injection of different compounds, sequentially, disrupts the ETC. Measurements taken after each injection allow for different mitochondrial parameters to be calculated, which include basal respiration, ATP turnover, maximal respiration, and spare capacity. After culturing CECs and exposing them to various concentrations of FCCP, basal respiration, ATP production, maximal respiration, and spare capacity were determined. Results indicate all four mitochondrial parameters were significantly decreased at doses of both 1000 nM and 10000 nM (p<0.0001), while there was no difference observed at the 10 nM and 100 nM exposures (Figure 1). These data suggest that uncoupling of the ETC decreases mitochondrial phosphorylation in CECs at 1000 nM or greater concentrations of FCCP.

### FCCP worsens stroke outcomes in mice

**Figure 2:**
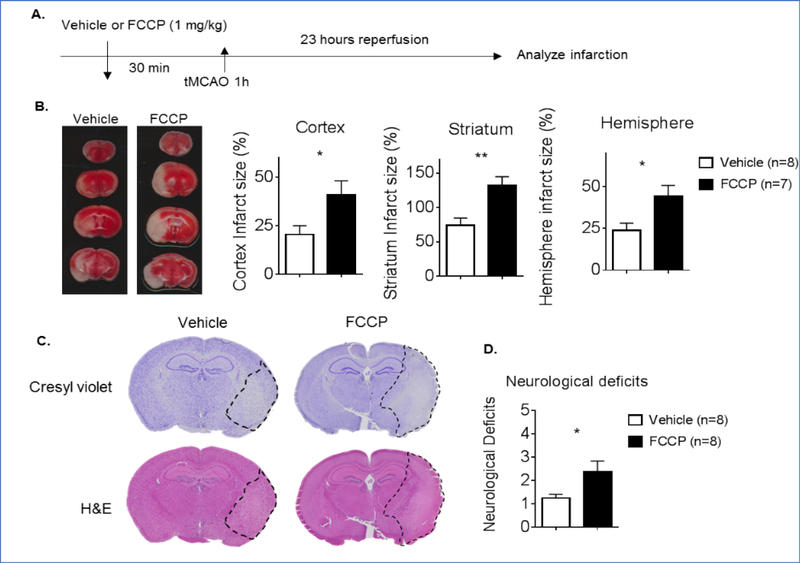
**(A-D)** Uncoupling the ETC with FCCP increased stroke infarction and neurological deficits in mice. (A) Mice were exposed to FCCP (1 mg/kg) or the vehicle 30 min prior to tMCAO (60 min) FCCP (n=8) compared to the vehicle (n=8). (B) TTC staining following 23 hr reperfusion. Pre-treatment with FCCP significantly increased infarct volume in the cortex, striatum, and total hemisphere when compared to the vehicle (*p<0.05 and ** p<0.01 respectively). (C) The Cresyl violet staining and H&E staining confirmed larger infarction in mice receiving FCCP outlined with a dashed line. (D) Mice receiving FCCP had a significantly increased neurological deficit score compared to the vehicle (*p<0.05). Bar graph data are presented as mean ± SEM.

In order to understand the impact of the uncoupling of the ETC might have during a stroke, animals were treated with either vehicle control or FCCP (1 mg/kg) at 30 min prior to tMCAO (Figure 2A). Following a 23 hr reperfusion, mice were sacrificed and TTC staining was completed. Results demonstrate that pretreatment of mice with FCCP prior to tMCAO significantly increased infarct volume when compared to the vehicle control. Increases in infarct size were observed in the cortex, striatum, and total hemisphere of FCCP treated mice (Figure 2B). Cresyl violet staining and H&E staining confirmed there was a larger infarction in mice receiving FCCP (Figure 2C). Mice receiving FCCP demonstrated increased severity on the neurological deficit scale (Figure 2D). The data suggests that the uncoupling of the ETC prior to a stroke may lead to exacerbated stroke infarction and neurological dysfunction.

## Discussion

The importance of maintaining mitochondrial function following an ischemic event in the brain has been well-demonstrated. Previous work revealed that loss of the mitochondrial uncoupling protein-2, which leads to inhibition of the ETC, led to an increased infarct volume of 61% per hemisphere compared to 18% in wildtype [14]. Other studies have found that low mitochondrial DNA content is associated with heightened effects of ischemic stroke [15]. In two other studies, the effects of LPS and miR-34a were shown to affect the integrity of the BBB, and in both of these studies, there was measurable mitochondrial dysfunction. Along with miR-34a and LPS effects, NAD-Dependent Protein Deacetylase Sirtuin-1 (SIRT1) may be another main player in mitochondrial dysfunction [16]. SIRT1 is known to play a crucial role in cancers, obesity, stroke, and dementia along with regulation of metabolism and the modulation of metabolic diseases [17–19]. Our previous study demonstrated that using rotenone, a Complex I inhibitor, compromised mitochondrial activity in a murine model, and this dysfunction, along with altered body temperature, led to poor outcome following a stroke [20]. In a study by Feng et al. [21], treatment with rotenone reduced SIRT1 levels and AMPK phosphorylation. Furthermore, miR-34a regulates SIRT1 [17], and miR-34a is related to mitochondrial dysfunction. However, it is not known whether FCCP in involved in the regulation of SIRT1- this could be an interesting topic to be addressed further. These studies provide evidence for the importance of maintaining mitochondrial function following an injury to the brain [22,23]. Our previous study demonstrated that the mitochondria inhibitor FCCP disrupts the integrity of the BBB and increases BBB permeability *in vitro* [17]. The data from our studies provides additional support that compromising the integrity of the ETC by using an uncoupling agent like FCCP leads to mitochondrial dysfunction in a dose-dependent manner. Pre-treatment of animals with FCCP 30 min prior to a tMCAO (60 min) led to a significantly larger infarct volume when compared to vehicle control.

Some limitations in our studies are derived from the fact that overall infarct size can be dependent on other factors such as ischemia duration and severity, sufficient blood pressure, age, comorbidities, genetic background, etc [24], and it will be important to consider these factors in order to determine the full effects that mitochondrial dysfunction may have on stroke outcome.

## Conclusion

In conclusion, our results demonstrate that the uncoupling of the electron transport chain compromises mitochondrial oxidative phosphorylation in CECs, observed by a decrease in spare capacity, ATP turnover, basal respiration, and maximal respiration. Chemically uncoupling the ETC exacerbated stroke infarction in a murine tMCAO model, with measurable increases in infarct volume within the cortex, striatum, and total hemisphere. Given the data from our studies, as well as previously reported work, it is increasingly apparent that maintaining and/or restoring mitochondrial function following an ischemic stroke is critical to minimize infarct damage. Future studies should address how to restore mitochondrial function and focus on development of therapeutics that could be given to patients following an ischemic stroke, with the idea that restoration of mitochondrial function and decreased infarct size will lead to better outcomes for stroke patients.
